# A Prospective Three-Year Cohort Study of the Epidemiology and Virology of Acute Respiratory Infections of Children in Rural India

**DOI:** 10.1371/journal.pone.0000491

**Published:** 2007-06-06

**Authors:** Shobha Broor, Shama Parveen, Preeti Bharaj, Velisetty S. Prasad, Kavalu N. Srinivasulu, Krishna M. Sumanth, Suresh Kumar Kapoor, Karen Fowler, Wayne M. Sullender

**Affiliations:** 1 Department of Microbiology, All India Institute of Medical Sciences, New Delhi, India; 2 Comprehensive Rural Health Services Project, Centre for Community Medicine, All India Institute of Medical Sciences, New Delhi, India; 3 Department of Pediatrics, University of Alabama at Birmingham, Birmingham, Alabama, United States of America; 4 Department of Epidemiology, University of Alabama at Birmingham, Birmingham, Alabama, United States of America; 5 Department of Maternal and Child Health, University of Alabama at Birmingham, Birmingham, Alabama, United States of America; 6 Department of Microbiology, University of Alabama at Birmingham, Birmingham, Alabama, United States of America; Medical University of South Carolina, United States of America

## Abstract

**Background:**

Acute respiratory infection (ARI) is a major killer of children in developing countries. Although the frequency of ARI is similar in both developed and developing countries, mortality due to ARI is 10–50 times higher in developing countries. Viruses are common causes of ARI among such children, yet the disease burden of these infections in rural communities is unknown.

**Methodology/Principal Findings:**

A prospective longitudinal study was carried out in children enrolled from two rural Indian villages at birth and followed weekly for the development of ARI, classified as upper respiratory infection, acute lower respiratory infection (ALRI), or severe ALRI. Respiratory syncytial virus (RSV), influenza, parainfluenza viruses and adenoviruses in nasopharyngeal aspirates were detected by direct fluorescent antibody testing (DFA) and, in addition, centrifugation enhanced culture for RSV was done. 281 infants enrolled in 39 months and followed until 42 months. During 440 child years of follow-up there were 1307 ARIs, including 236 ALRIs and 19 severe ALRIs. Virus specific incidence rates per 1000 child years for RSV were total ARI 234, ALRI 39, and severe ALRI 9; for influenza A total ARI 141, ALRI 39; for INF B total ARI 37; for PIV1 total ARI 23, for PIV2 total ARI 28, ALRI 5; for parainfluenza virus 3 total ARI 229, ALRI 48, and severe ALRI 5 and for adenovirus total ARI 18, ALRI 5. Repeat infections with RSV were seen in 18 children.

**Conclusions/Significance:**

RSV, influenza A and parainfluenza virus 3 were important causes of ARI among children in rural communities in India. These data will be useful for vaccine design, development and implementation purposes.

## Introduction

Acute respiratory infections (ARI) result in ∼1.9 million childhood deaths per year in developing countries, 20% of these deaths are in India [1], [2]. The role of viruses in the etiology of ARI in developing countries like India is not well studied. Among children hospitalized with acute lower respiratory infections (ALRI) in India, one half the infections are of viral etiology [3]–[5].

There have been no reports of longitudinal community based studies of viral ARI in India in the past 30 years [6]. The present study was undertaken to describe the epidemiology of ARI due to viruses among young children in two rural villages in India. The development of a capacity for prospective longitudinal studies of ARI in rural India should serve as a valuable resource of investigations of ARI, risk factors for ARI, and evaluation of interventions to reduce ALRI disease burden.

## Methods

### Study Population

Ballabgarh is a rural region in Haryana state in north India near Delhi. The population of over 70,000 is served by the Ballabgarh Primary Health Centre (PHC) under the Comprehensive Rural Health Services Project of All India Institute of Medical Sciences (AIIMS). The computerized records of all families in the communities are available at the PHC. For this study newborns were enrolled from two villages in Ballabgarh, Nwadha and Mujeri, with approximate populations of 2,300 and 1,700 respectively and birth rates of approximately 25 and 26/1000 respectively. The infant mortality rate for Ballabgarh was about 37 per thousand live births in 1997 [7]. The population of the villages is generally stable with very little migration. Newborns were identified from records available at the PHC and parents were asked to enroll their infants in the study. Informed consent was obtained from the parents in Hindi. Institutional review boards in India (Ethics Committee at All India Institute of Medical Sciences) and the USA (Institutional Review Board for human use [IRB] at University of Alabama at Birmingham) approved the study.

### Study follow-up and data collection

All information obtained was entered onto standardized proformas. Demographic data was collected by an interview with the parents and included child and birth characteristics; education and occupations of parents; and income and material possessions. Prematurity was defined as birth at less than 37 completed weeks of gestation [8].

### Monitoring for ARI

The fieldworkers visited the households weekly to identify ARIs in enrollees until the children were 3 years of age or the study ended. The field workers were trained to use WHO definitions for the classification of ARI [9]. Medical officers (physicians) supervised and provided ongoing training of the field workers.

### Definitions and Management of ARI

ARI was defined as cough or difficult breathing within the previous 7 days or observed during the visit. Each episode of ARI was considered to last two weeks, a new episode was counted if new signs and symptoms develop after the child has been symptom free for at least one week. The medical officers examined children with ARI/ALRI. Age adjusted definitions of fast breathing were as follows: age less than 2 months, 60 breaths per minute or more; age 2 months up to 12 months, 50 breaths per minute or more; and age 12 months up to 3 years, 40 breaths per minute or more. Danger signs (the presence of any of these signs required urgent referral to the hospital) were: inability to drink or breastfeed, child vomits everything, child has had convulsions, or child is lethargic or unconscious [9].

#### Children 2 months to 3 years of age

Chest indrawing or stridor in calm child or a child exhibiting any general danger sign was classified as severe ALRI or very severe disease. Treatment included giving the first dose of an appropriate antibiotic if available and referring the child urgently to the hospital in Ballabgarh. Fast breathing was classified as ALRI. Treatment included giving the appropriate oral antibiotic for 5 days and advising the mother as to when to go to clinic immediately. If there were no signs of ALRI or severe ALRI, classification was URI.

#### Children less than 2 months of age

Any general danger sign or fast breathing, chest indrawing, nasal flaring or grunting; classification severe ALRI or very severe disease. These children were referred urgently to the hospital. If these signs were not present classified as URI.

### Specimen Collection

At each ARI, a nasopharyngeal aspirate (NPA) was obtained by the medical officer. Specimens were collected through an infant feeding tube placed in the posterior nares and into a mucous trap using suction from a hand vacuum pump (Nalge Nunc Inter., Rochester, NY, USA). Cold viral transport media was aspirated into the mucous trap containing the NPA, the sample was placed on ice and transported to the Virology Laboratory at AIIMS within 6 hours [5].

### Virus Identification

Samples were processed and viruses were identified using antigen detection [10]. β-mercaptoethanol (100 µl of 2%) was added and the sample was mixed. Centrifugation was performed and the pellet was washed and resuspended in phosphate buffered saline. Smears from the cell suspension were placed on Teflon coated slides for DFA. Initial screening for presence of viral antigens used the SimulFluor® Respiratory Screen (Chemicon International, Inc.; Temecula, CA, USA). If RSV antigens were present golden fluorescence was observed, with other respiratory viruses (influenza A or B, parainfluenza 1, 2, 3 or adenovirus) green fluorescence resulted [11]. Samples positive by SimulFluor were tested by DFA Kit (Chemicon International) using individual monoclonal antibodies against RSV, influenza A (INF A) and B (INF B), parainfluenza 1 (PIV 1), 2 (PIV 2), and 3 (PIV 3), and adenovirus. Positive and negative controls were included. Positive identification by DFA required positive and congruent results by both SimulFluor and individual monoclonal testing by DFA. All slides with positive immunofluorescence results were reviewed by the senior investigator in charge of the Diagnostic Virology Laboratory at AIIMS (SB). Supernatants from the clarified samples were used for isolation of RSV by CEC [5] and presence of RSV was detected by indirect immunofluorescence staining with a blend of monoclonal antibodies that detects both group A and group B RSV (Chemicon International). Cultures were not performed for other viruses.

### Data entry and Analysis

The data for the study were entered into an Epi Info 2000 software database with customized views containing suitable range and consistency checks to prevent invalid data from being entered. All data were double keyed into the database. The overall incidence rates (IR) of ARI, ALRI and severe ALRI were determined by dividing the sum of new episodes by the total number of child years at risk and presented as cases per 1000 child-years. Child years were calculated from the date of enrollment until either the child died, refused further follow up, reached 3 years of age, or through March 30, 2005. Confidence intervals (95%) for IRs were calculated by exact binomial methods.

## Results

### Characteristics of study population

Population characteristics were similar between the villages Nwadha and Mujeri and the composite data are presented. All of the mothers were married and indicated their occupation as housewife. Many of the mothers were illiterate (46%), their age was 23±3.8 years (mean±SD). Fathers were employed as day laborers (21%), farmers (26%) small business owners (25%), or were employees of business or government (28%). Reported median incomes were 36,000 (range 5,000–144,000) rupees (∼USD $777, range $108–$3,107) per year. Births were almost equally divided between home (47%) and in a hospital or clinic (53%) and 11% were preterm.

### Enrollments in the study

The enrollment began October 1, 2001 and ended after 39 months (December 31, 2004), follow-up concluded after a total study period of 42 months (March 31, 2005). Enrollment was almost equally divided between the two villages (Nwadha 145, Mujheri 136). A total of 281 infants were enrolled, including 148 males and 133 females accounting for 86% of the 325 births in this period. Twenty-two children enrolled and then opted out of the study and six deaths occurred, none were due to ARI. The age at enrollment ranged from 1 day to 10.7 months (mean±SD, 1.4±1.7) with a median of 0.8 months. The period of follow up ranged from 1 month to 36 months (mean±SD, 20.4±10.4) and a median of 20 months. Accounting for deaths and drop-outs there were 440 child-years of follow-up.

### ARIs in study population

A total of 1307 ARIs were recorded of which URIs accounted for 1052 (80%), ALRIs 236 (18%), and severe ALRIs 19 (1.5%). Incidence rates by disease category and age are given (Table 1), there were 2966 ARIs, including 536 ALRI and 43 severe ALRI per 1000 child years (cy). By age group severe ALRI was most common in infants (62 per 1000 cy) and 85% of these occurred in infants less than 6 months of age. The rates for ALRI were highest in the 12–23 month age group at 831 per 1000 cy, as compared to 389 for 24–35 months and 370 for 0–11 months. Of the 281 children enrolled 268 (95%) had between one and 15 episodes of ARI.

**Table 1:** Incidence rates of ARIs in the study population by age

Age (months)	Total ARIs IR (95%CI)	URI IR(95% CI)	ALRI IR (95% CI)	Severe ALRI IR (95% CI)
0–11	3504 (3264–3756)	3073 (2847–3311)	370 (293–461)	62 (33–106)
12–23	2952 (2694–3226)	2094 (1877–2330)	831 (695–986)	26 (7–66)
24–35	1474 (1215–1772)	1059 (840–1316)	389 (261–557)	27 (3–97)
Total	2966 (2812–3126)	2387 (2248–2532)	536 (470–608)	43 (26–67)

Note. Incidence rate (IR) per 1000 child years and 95% Confidence Intervals (CI)

### Virus identification by direct fluorescence assay

Nine hundred forty four samples were sufficient for analysis (adequate cell numbers and not obscured by mucus) by DFA of 1279 NPAs collected. A total of 233 respiratory viruses were identified by DFA (Table 2) in 147 samples. Interestingly, 62 samples revealed the presence of antigens from more than one virus. PIV 3 and INF A was the most common pair of viruses identified in the mixed infections. PIV3 and INF A were also the most commonly identified viruses, with 101 PIV3 and 62 INF A positive samples when mixed infections were included. As single agents, PIV3 was identified in 46 and INF A in 11 instances. RSV was found in 24 samples, as a single agent in 14.

**Table 2:** DFA for detection of respiratory viruses in ARI

Viruses	Positive
RSV alone	14
INF A alone	11
INF B alone	3
PIV 1 alone	0
PIV 2 alone	8
PIV 3 alone	46
Adeno alone	3
RSV, INF A	3
RSV, INF A, PIV 3	3
RSV, INF A, PIV 2	1
RSV, INF A, INF B, PIV 3	1
RSV, PIV 3	2
INF A, PIV 3	28
INF A, PIV 2	1
INF A, PIV 1	1
INF A, INF B, PIV 3	3
INF A, PIV 1, PIV 3	3
INF A, PIV 3, Adeno	1
INF A, INF B, PIV 2, PIV 3	1
INF A, INF B, PIV 1, PIV 3	1
INF A, PIV 1, PIV 2, PIV 3	1
INF B, PIV 3	5
INF B, PIV 1	1
INF B, PIV 1, PIV 3	1
PIV 1, PIV 3	1
Adeno, PIV 3	1
Adeno, INF A, PIV 3	2
Adeno, INF A, PIV 1, PIV 3	1
**Total samples (+) for viruses**	**147**

### Centrifugation enhanced culture identification of RSV

RSV was detected in 95 of 1279 samples tested by CEC (7.4%). One hundred and three (8%) samples were positive for RSV by one or both assays (DFA and CEC). In 944 samples tested by both DFA and CEC, RSV was detected in 25 (2.5%) by DFA and 75 (7.9%) by CEC. Of these, 13 were positive by both assays, 11 by DFA alone and 62 by CEC alone. Of the 86 samples positive for RSV by one or both assays 87% (75/86) were detected by CEC and 28% (24/86) by DFA. Thus, sensitivity of RSV detection was increased by use of both the techniques, DFA and CEC [5]. Some of the RSV strains underwent molecular characterization [12], [13].

### Distribution of viruses by age and severity of illness

RSV, INF A, and PIV 3 infection incidence rates (IR) are shown by disease category and age (Table 3). The RSV total ARI IR was highest in the first year of life (322 per 1000 cy). The RSV associated ALRI IR was actually higher in the 12–23 months group as compared to other ages, whereas for RSV severe ALRI the IR was slightly higher in the 0–11 month as compared to the 12–23 month group. RSV was found in 8% of URIs, 7% of ALRIs, and 21% of severe ALRIs. RSV was identified by DFA and CEC, for the other viruses only DFA was performed (944). Thus, IRs cannot be directly compared between RSV and the other virus types. INF A total ARI IR was 141 per 1000 cy, the IR for ALRI was 39, and no severe ALRIs were identified. INF A IR were similar for 0–11 and 12–23 months and slightly lower in the 24–35 month group. INF A was found in 6% of URIs and 11% of ALRIs. The PIV3 total ARI IR was 229 per 1000 cy, with ALRI 48 and severe ALRI 5 per 1000 cy. In the first 2 years of life the IR for PIV3 was very similar, but fell in the third year. PIV 3 was found in 10% of URIs, 11% of ALRIs, and 15% of severe ALRIs.

**Table 3:** Severity of ARI and incidence rates for viruses by age groups

	RSV IR (95% CI)				INF A IR (95% CI)				PIV 3 IR (95% CI)			
	0–11	12–23	24–35	all ages	0–11	12–23	24–35	all ages	0–11	12–23	24–35	all ages
**URI**	275 (211–351)	122 (74–188)	67 (22–150)	186 (149–229)	114 (73–168)	97 (55–157)	80 (30–167)	102 (75–136)	223 (165–293)	149 (95–218)	107 (47–201)	177 (141–219)
**ALRI**	33 (14–65)	52 (23–98)	27 (3–89)	39 (23–60)	33 (14–65)	58 (27–107)	13 (0–69)	39 (23–60)	38 (17–71)	77 (41–130)	13 (0–69)	48 (30–71)
**Severe ALRI**	14 (3–33)	7 (0–21)	0	9 (3–20)	0	0	0	0	5 (1–166)	7 (0–21)	0	5 (0–14)
**Total**	322 (253–404)	180 (121–257)	94 (38–187)	234 (192–281)	147 (100–207)	155 (100–227)	94 (38–187)	141 (108–179)	265 (202–341)	232 (164–316)	121 (56–221)	229 (188–276)

Note. RSV detection by DFA and CEC, INF A and PIV 3 detected by DFA alone. Incidence rate (IR) per 1000 child years.

INF B, PIV1 and 2, and adenovirus are not shown because they were identified rarely in children with ALRI and never from children with severe ALRI. The total IRs were as follows: INF B 37, PIV1 23, PIV2 28, and adenovirus 18 per 1000 cy. INF B and PIV1 were not identified in ALRIs. The IR for ALRI for PIV 2 was 5 and for adenovirus was 5 per 1000 cy.

### Monthly distribution of viruses

**Figure 1:**
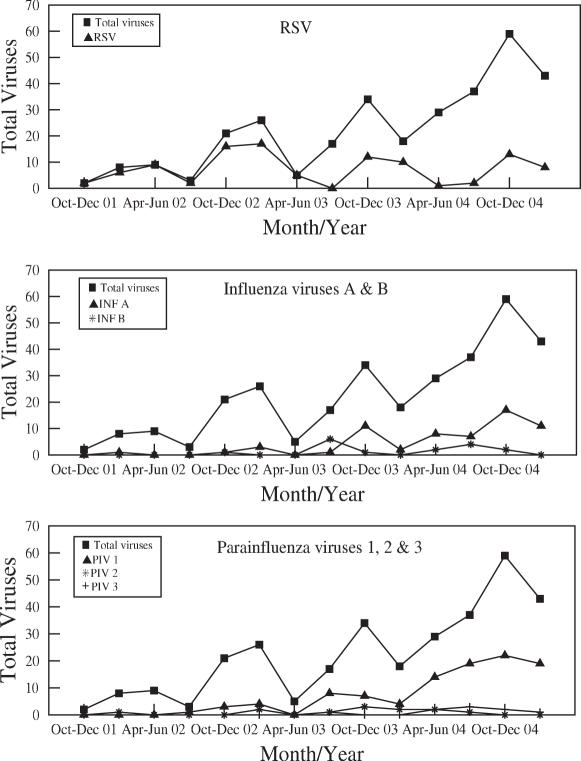
Monthly distribution of viral identification throughout study period October 2001 to March 2005. RSV was identified by DFA and CEC, INF and PIV by DFA. Total positive represents the total number of samples with a virus identified. Panel A, RSV; panel B, influenza A and B; panel C, parainfluenza 1, 2, and 3.

The monthly distribution of viruses is shown (Figure 1). RSV identifications were most frequent in the fall and winter but continued at a lower level throughout the year with the exception of the rainy season of July and August. During the fall and winter 2004–2005 much more PIV 3 and INF A activity was noted as compared to the previous years and these viruses were found more often than RSV in this epidemic period.

### Repeat Infections with RSV

RSV primary infections were found in 30% (83/281) and 18 children (6.4%) had one or more reinfections, 14 children with one and 4 with two documented reinfections (Table 4). Thus, there were 22 RSV identifications in reinfections (50 per 1000 cy), accounting for 21% (22/103) of all RSV identifications. The repeat infections occurred 1–27 months after the previous infection. The ages of children at which the first RSV infection was detected ranged from 2–17 months, at second infection was 3–26 months and at third infection was 15–30 months. Twelve repeat infections were identified in same season and ten reinfections in different RSV seasons. Twelve patients had URI during both first and second infections and three patients had URI during first, second and third infections. Among these 18 patients, ALRI occurred in three reinfections. There were no severe ALRI identified as repeat RSV infections.

**Table 4:** Demographics and disease severity with repeat infections by RSV

Patient	Age in Months	Reinfection Interval in Months	Viruses detected	ARI Classification
1	12		RSV&INF A	URI
1	24	12	RSV	URI
2	7		RSV	URI
2	26	19	RSV	ALRI
3	5		RSV, INF A, PIV 1&PIV 3	URI
3	9	4	RSV	URI
4	17		RSV	URI
4	20	3	RSV	URI
4	23	3	RSV	URI
5	9		RSV	URI
5	15	6	RSV, INF A&PIV 3	URI
6	2		RSV	URI
6	18	16	RSV	URI
7	5		RSV	URI
7	6	1	RSV	URI
8	3		RSV	URI
8	12	9	RSV, INF A&PIV 2	URI
9	4		RSV	URI
9	7	3	RSV	URI
9	15	8	RSV	URI
10	5		RSV	URI
10	12	7	RSV	ALRI
11	2		RSV	URI
11	7	5	RSV	URI
12	2		RSV	URI
12	5	3	RSV	URI
13	2		RSV	URI
13	3	1	RSV	URI
13	30	27	RSV	URI
14	3		RSV	URI
14	5	2	RSV	URI
15	2		RSV	URI
15	12	10	RSV, INF A&PIV 3	URI
16	5		RSV	URI
16	17	9	RSV	ALRI
16	19	2	RSV	URI
17	14		RSV	URI
17	15	1	RSV	URI
18	3		RSV	URI
18	10	7	RSV&PIV 3	URI

Note: virus detection by DFA or, for RSV, DFA and CEC.

ARI classification of disease per WHO [9].

## Discussion

Globally, RSV may cause as many as 0.5 million deaths in children each year [14]. In developing countries relatively few studies have been performed of the viral causes of ARI outside the setting of urban hospitals [15]. This is true for India, the second most populous country in the world. In addition, 70% of the population of India is rural, and urban hospital results cannot be assumed to represent ARI epidemiology for the entire population [16]. In this report we describe a prospective, longitudinal, community based investigation of viral ARIs in children of India. The study population was rural and of lower socioeconomic status and in these aspects is typical of the majority of the Indian population.

The incidence rates in the present study are comparable to those recently reported from other developing countries. The total ARI incidence rate of 2966 per 1000 cy was similar to the 3700 previously reported from the same region [1]. The IR per 1000 cy for ALRI were 600 in Philippines [18] and 536 in the present study. Other developing countries either reported either lower incidence rates with 100 in Thailand [18], 191 in Indonesia 270 in Nigeria [17] or higher with 4200 in Uruguay and 1800 per 1000 cy in Colombia [18]. In the current study higher rates of ALRI were observed in the second year of life (831), as compared to the first year (370) or third year (389). The highest rates of ALRI generally occur in the first year of life. However, in the BOSTID studies the Philippines and Colombia reported higher ARI rates in the 12–23 as compared to the 0–11 months groups, and the ALRI rates in the Philippines and Guatemala were similar for the two age groups [18]. The severe ALRI rate observed in the first year of life, 62 per 1000 cy, falls with-in the range of values from other developing countries, including Indonesia 25, Mozambique 126, and South Africa 332 per 1000 cy [17].

RSV primary infections were found in 30% of the children. A prospective study in Houston, Texas used a combination of virus isolation and serology and found that by 12 months of age 68% and by 24 months of age 97% of children had been infected with RSV [19]. Unless the risk of RSV infection is substantially lower in India as compared to the Texas study it seems likely additional RSV infections occurred that were not detected. Thus our data represent a minimal estimate of the burden of RSV disease for this population of rural Indian children. The total RSV ARI results (234 per 1000 cy) may be compared to those reported from Kenya, (428 per 1000 cy) [20]. However, RSV ALRI and severe ALRI were much higher in Kenya. This may be due to the inclusion of children from the local health clinic along with an active surveillance component in the Kenya study. In the present study the IR for RSV associated ALRI was 39 and severe ALRI was 9 per 1000 cy. These results may be compared to those from a WHO sponsored study, with RSV associated ALRI in Indonesia 34 and Nigeria 94 per 1000 cy. The rate of RSV associated severe ALRI in Indonesia was 10 and Mozambique 5 per 1000 cy [17]. In the study reported here RSV associated disease was much more common in the first year of life as compared to the older children, for INF A and PIV 3 the first and second years of life had similar IRs. The similarities among the results from the different studies are more striking than the differences considering the diverse populations investigated and the differences in definitions, study design and surveillance, and viral detection methods used.

Almost 16% of (147/944) of samples tested by DFA were positive for viruses. Interestingly, 42% of the samples revealed the presence of more than one viral antigen. Mixed infections have been reported in other studies. A study of hospitalized children from New Zealand that used RT-PCR for virus detection found multiple infections in 27% [21]. In another study dual infections with influenza and parainfluenza virus were reported in 35% of the children hospitalized for viral acute respiratory illnesses [22]. Additional efforts, including viral culture and application of molecular detection techniques, will be important in the explanation of the mixed infections identified by DFA.

Most virus identifications occurred in the fall and winter as expected from a temperate climate [5]. A 1964–1969 study in rural villages outside tropical Calcutta found RSV primarily during September and October, following the monsoon season [23]. The same group found PIV identifications exceeded that of RSV, with PIV in 11% and RSV in 2% of children in the years 1964–1966 [6]. In the current study the 2004–2005 epidemic period was notable for the contribution of both INF A and PIV 3 to the viral ARI disease burden.

Reinfections with RSV are the norm and occur throughout life. In this study the IR for reinfections was lower (50 per 1000 cy) than that reported from Kenya (192 per 1000 cy) [20]. In 3 children repeat infections were ALRI, thus primary infection did not protect against later lower respiratory disease. This is in agreement with earlier work that found a single infection may not be sufficient to reduce illness upon reinfections [24]. A subset of the viruses presented here underwent detailed molecular characterization [13]. In this study and in work done in Kenya viruses belonging to the same genetic groups or lineage or even apparently identical viruses have been detected in reinfections [25]. The role of antigenic diversity in the establishment of reinfections requires further investigation.

This is the first community-based study of viral ALRI in India in more than three decades [6]. The results show that RSV, INF A, and PIV3 are important causes of ARI, including ALRI, among young children. Severe ALRI was more commonly caused by RSV than by INF A or PIV 3. Because standard WHO definitions were used and a stable cohort was followed prospectively these data include denominator information typically unavailable from hospital-based studies. These data will be useful for planning the study of future respiratory virus vaccines or other interventions to reduce the disease due to viral ALRIs. However, additional studies with a larger population will be required to more precisely define viral ARI disease burden.
